# Colon Cancer Presenting as Pituitary Mass and Hypopituitarism: Recognition and Multidisciplinary Approach of a Rare Case

**DOI:** 10.1210/jcemcr/luad031

**Published:** 2023-04-21

**Authors:** Flavia Costanza, Antonella Giampietro, Pier Paolo Mattogno, Sabrina Chiloiro

**Affiliations:** Pituitary Unit, Division of Endocrinology and Metabolism, Fondazione Policlinico Universitario A. Gemelli IRCCS, Rome, 00168, Italy; Department of Translational Medicine and Surgery, Catholic University of Sacred Heart, Rome, 00168, Italy; Pituitary Unit, Division of Endocrinology and Metabolism, Fondazione Policlinico Universitario A. Gemelli IRCCS, Rome, 00168, Italy; Department of Neurosurgery, Fondazione Policlinico Universitario A. Gemelli IRCCS, Rome, 00168, Italy; Pituitary Unit, Division of Endocrinology and Metabolism, Fondazione Policlinico Universitario A. Gemelli IRCCS, Rome, 00168, Italy; Department of Translational Medicine and Surgery, Catholic University of Sacred Heart, Rome, 00168, Italy

**Keywords:** pituitary metastases, gastrointestinal carcinoma, hypopituitarism, adrenal insufficiency

## Abstract

Pituitary metastases are rare. Until now, few cases have been reported; about 50% of pituitary metastases originate from breast or lung cancers. We describe the clinical case of a primary colon carcinoma first presenting with a pituitary metastasis. A 76-year-old woman, with no history of malignancy, presented with headache, dizziness, and diplopia, at the Emergency Department. The neurologic examination was remarkable for complete left ophthalmoplegia with sensitivity deficit on the left side of the face. Radiologic investigations documented a voluminous sellar and suprasellar lesion, with extension in the left cavernous sinus and temporal lobe. Pituitary hormone levels were suggestive of anterior hypopituitarism and mild hyperprolactinemia. Subtotal surgical removal of the lesion was achieved through a trans-sphenoidal endoscopic endonasal approach. The histological examination disclosed a metastasis of gastrointestinal adenocarcinoma. A subsequent colonoscopy identified right colon cancer. A contrasted total-body computerized tomography ruled out other metastases. Postsurgical MRI showed a stable parasellar residual tumor. Conventional radiotherapy was scheduled.

This case underlines the importance of considering pituitary metastases in the differential diagnosis of aggressive pituitary lesions, which should be managed in a pituitary tumor center of excellence through a multidisciplinary approach, for the complexity in diagnosis and therapeutic management of this rare condition.

## Introduction

Pituitary metastases are rare. Few cases are reported in the literature. The current incidence is about 1%, without gender difference [1]. Autopsy studies proved a prevalence between 1-4% in patients with advanced cancer [1]. Pituitary metastases occur most often between 60 and 70 years [1]. Pituitary metastases originate from breast and lung cancers, in about 50% of cases, and more rarely from other sites such as thyroid, prostate, kidney, liver, melanoma, bladder, gastrointestinal and blood neoplasia [2].

Pituitary metastases may rapidly grow and invade the surrounding vascular and nerve structures, leading to pituitary dysfunctions, such as hypopituitarism and diabetes insipidus, and to neurological symptoms, such as headache, visual acuity or campimetric deficit, or cavernous sinus syndrome [3]. The cavernous sinus syndrome may be caused by an expansive/infiltrative mass that extends into the cavernous sinus and disrupts the function of other anatomical structures, such as vessels and cranial nerves, inducing ophthalmoplegia, ptosis, and facial sensory loss.

Pituitary metastases are considered life-threatening conditions, particularly in cases of sudden occurrence of central adrenal insufficiency that requires a prompt diagnosis and treatments to reduce the risk of occurrence of adrenal crisis. Pituitary metastasis may impact the therapeutic management of patients, in particular in those with no history of cancer.

To our knowledge we report the first case of a patient with pituitary metastasis of a primary gastrointestinal adenocarcinoma, first presenting as pituitary mass in a patient with no cancer history.

## Case Presentation

A 76-year-old woman arrived in the Emergency Department for asthenia, headache, dizziness, and diplopia in February 2022. The patient was affected by systemic arterial hypertension on treatment with beta-blockers and by multinodular thyroid goiter on treatment with 75 micrograms of levothyroxine daily. The patient had no history of previous or active oncological disease. At admission, she denied nausea, vomiting, fever, polyuria, and polydipsia. The vital parameters were: body temperature of 36.2 °C, systemic blood pressure of 135/80 mmHg, heart rate of 84 bpm. The neurologic examination showed complete left ophthalmoplegia and sensitivity deficit on the left side of the face.

## Diagnostic Assessment

**Figure 1.**
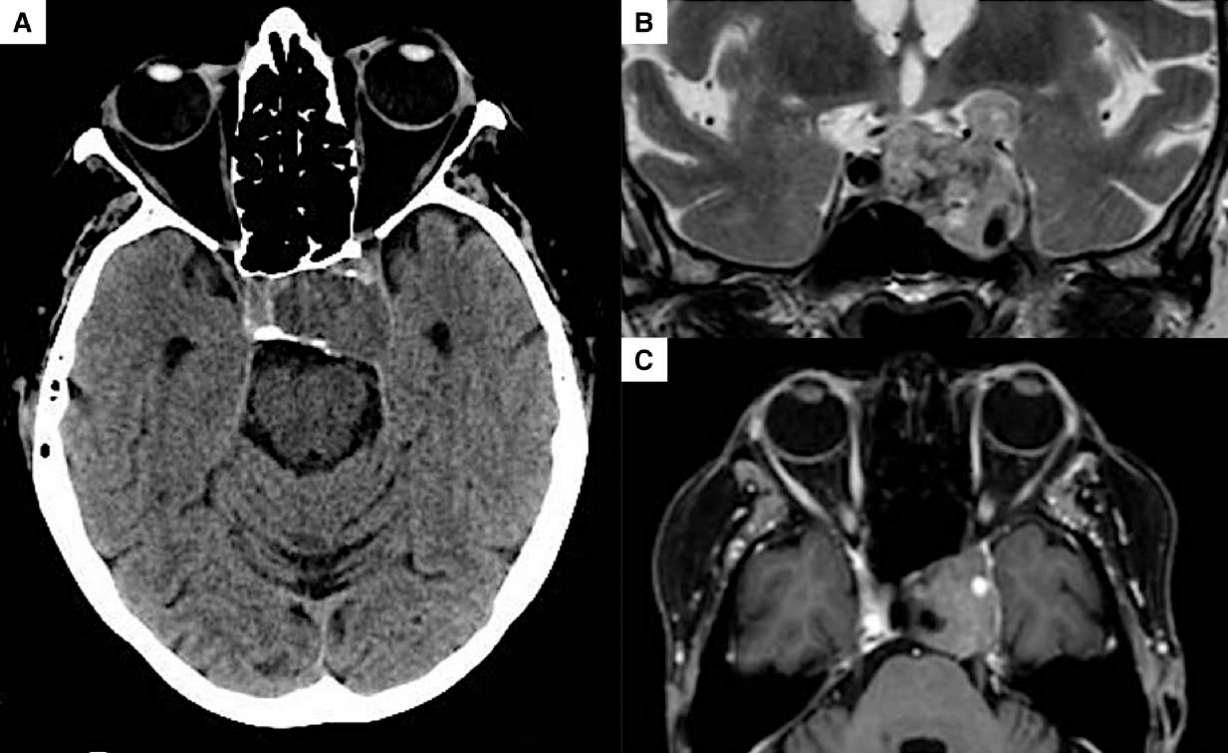
A, Axial CT image of sellar-parasellar mass, without contrast medium. B, and C, Coronal T2 weighted image and axial T1 postcontrast image of sellar-parasellar mass, with left cavernous sinus invasion and extension to temporal lobe.

A brain computed tomography (CT) showed a sellar-parasellar mass (maximum diameter: 30 mm), with invasion of the sphenoid and of the left cavernous sinus, encasement of the intra-cavernous internal carotid artery (ICA) and compression on the left temporal lobe (Fig. 1A).

A subsequent contrasted magnetic resonance (c-MR) proved a heterogeneous contrast enhanced sellar lesion, suggestive of intra-lesion hemorrhage and apoplectic infarction (Fig. 1B and 1C). Left cavernous sinus syndrome in apoplectic macro-adenoma was suspected.

Hormone test results in anterior hypopituitarism with mild hyperprolactinemia, according to inappropriate low follicle-stimulating hormone (FSH), luteinizing hormone (LH), thyroid-stimulating hormone (TSH), and adrenocorticotropic hormone (ACTH) levels, as shown in Table 1. The patient did not refer polyuria and polydipsia. Serum sodium concentration was 148 mEq/L, with a blood osmolarity of 285 mOsm/L. Urinary specific gravity was 1.025.

**Table 1.** Presurgery and postsurgery pituitary hormone values according to conventional and SI Units

	Presurgery hormone test		2 days postsurgery hormone test		15 days postsurgery hormone test	
	Conventional units	SI unit	Conventional units	SI unit	Conventional units	SI unit
TSH	0.5 microUI/mL (range, 0.35–3.80)	0.5 mIU/L	0.06 microUI/mL (range, 0.35–3.80)	0.06 mIU/L	Not available	
fT4	9.8 pg/mL (range, 8.5–16.5)	12.6 pmol/L	8.7 pg/mL (range, 8.5–16.5)	11.2 pmol/L	Not available	
fT3	1.4 pg/mL (range, 2.4–4.2)	2.15 pmol/L	1.4 pg/mL (range, 2.4–4.2)	2.66 pmol/L	Not available	
ACTH	< 5 pg/mL (range, 10–55)	<1.1 pmol/L	5 pg/mL (range, 10–55)	1.1 pmol/L	15 pg/mL (range, 10–55)	3.3 pmol/L
Cortisol	80 ng/mL (range, 60–220)	220.7 nmol/L	84 ng/mL (range, 60–220)	231.7 nmol/L	120 ng/mL (range, 60–220)	331 nmol/L
Prolactin	110 microUI/mL (range, 3.5–26)	5.17 microgr/L	20 microUI/mL (range, 3.5–26)	0.94 microgr/L	Not available	
GH	0.3 ng/mL	0.3 microg/L	0.4 ng/mL	0.4 microg/L	Not available	
IGF-1	55 ng/mL (range, 33–212)	7.2 nmol/L	73 ng/mL (range, 33–212)	9.5 nmol/L	Not available	
LH	<0.1 mIU/mL (range, 2.5–15 in menopause age)	<0.1 IU/mL	mIU/mL (range, 2.5–15 in menopause age)	0.1 IU/mL	Not available	
FSH	mUI/mL (range, 22–116 in menopause age)	0.1 IU/L	0.03 mUI/mL (range, 22–116 in menopause age)	0.03 IU/L	Not available	
Estradiol	15 pg/mL (range, <25 in menopause age)	55 pmol/L	8 pg/mL (range, <25 in menopause age)	29.4 pmol/L	Not available	

Abbreviations: ACTH, adrenocorticotropic hormone; fT3, free triiodothyronine; fT4, free thyroxine; FSH, follicle-stimulating hormone; GH, growth hormone; IGF-1, insulin-like growth factor 1; LH, luteinizing hormone.

## Treatment

A transsphenoidal endoscopic endonasal surgery was conducted to remove the pituitary mass. Following our institutional peri-surgery protocol, intravenous 100 mg hydrocortisone was administered at the induction of anesthesia, and after surgery every 8 hours, on the first postoperative day, according to the hemodynamic conditions of the patient. No postsurgical complications occurred. A mild ophthalmoplegia persisted in the left eye.

According to the patient's clinical condition and to the pituitary hormone levels that were conducted 2 days after surgery (Table 1), we prescribed hormonal replacement therapy with hydrocortisone 25 mg/daily and levothyroxine 100 micrograms/daily, at the discharge from the neurosurgery department. An emergency card for the management of the adrenal crisis was released to the patient and her relatives.

At 15 days follow-up, hormone replacement therapy with hydrocortisone was discontinued, according to the improvement of the patient's clinical condition, due to the resolution of asthenia and dizziness and to the improved ACTH-cortisol secretion, as shown in Table 1.

The histological examination disclosed a necrotic and hemorrhagic tissue, with a trabecular microarchitecture, epithelial cells, with round and often irregular nuclei and large and vacuolated cytoplasm. The immunohistochemical profile was negative for synaptophysin, pituitary-specific positive transcription factor 1 (PIT-1), GATA-3, T box transcription factor (T-PIT) and the pituitary hormones: ACTH, growth hormone, prolactin, LH, FSH, TSH.

The tumor cells expressed the caudal-type homeobox 2 (CDX-2) and the cytokeratin (CK): CK-7, CK-20 and did not express the Paired Box 8 (PAX8) and the anti-thyroid transcription factor (TTF-1). The histological examination was suggestive of a pituitary metastasis of an adenocarcinoma with a possible gastrointestinal origin. A whole-body CT was performed, detecting a suspect pathological thickening of the right colon. The CT did not prove expansive or infiltrative lesions of lung, liver, pancreas, adrenal glands, and bones. In parallel, no abdominal, ilo-mediastinal, supraclavicular, axillary, and neck lymphadenopathies were detected.

A colonoscopy was performed in a different institution and detected right colon cancer. The patient underwent a surgical removal of the lesion.

## Outcome and Follow-up

Three months after surgery, a brain MRI showed a parasellar residual of the pituitary metastasis, which was radiologically stable at 6 months follow-up. Conventional radiotherapy was scheduled, according to institutional multidisciplinary evaluation that involved oncologists, radiation oncologists, neurosurgeons, neuroendocrinologists, neuroradiologists, and neuropathologists.

## Discussion

Pituitary metastases from gastrointestinal cancers were rarely described; about 50% of pituitary metastasis originates from breast or lung cancers and around 3% to 5% are from kidney, prostate, melanoma, thyroid, pancreas, blood, and from unknown primary cancers [1].

To our knowledge, our clinical case reports for the first time the history of a patient with pituitary metastasis of a primary gastrointestinal adenocarcinoma, presenting as pituitary mass in a patient with no cancer history.

The diagnosis of pituitary metastasis from primary gastrointestinal adenocarcinoma was supported by the immunohistochemical positivity for CDX-2, CK-7, and CK-20 [4].

The subsequent identification of right colon cancer with the same immunohistochemical profile of those of the pituitary mass confirmed the diagnosis of pituitary metastasis.

Pozzessere et al described a patient with synchronous pituitary and liver metastases from gastric cancer, that were diagnosed after the identification of the primary tumor. Similarly, Issa et al described a case of a stage III colon adenocarcinoma that was diagnosed 4.5 years before the occurrence of the pituitary metastasis. In our case, the patient had no cancer history.

Pituitary metastases typically occur with acute neurological symptoms [5]. Partial or complete hypopituitarism and diabetes insipidus can occur for the destruction of the gland but also for the mass effect, and may represent an emergency, in particular in cases of central adrenal insufficiency [3].

According to Schill et al, the increased prevalence of ACTH deficiency in patients with malignancies highlights the importance of considering cancer dissemination to the pituitary.

The occurrence of new-onset fatigue, nausea, and loss of appetite should lead to prompt screening for hypocortisolism. Glucocorticoid replacement and correction of other pituitary dysfunctions may also improve the tolerance to chemotherapy [6].

Symptomatic pituitary metastasis can represent the first clinical manifestation of cancer and can closely mimic a pituitary adenoma [7]. Despite previous literature suggesting that the most common sign of pituitary metastasis was diabetes insipidus [8], an increased incidence of anterior hypopituitarism was more recently reported, also with a deeper study of the pituitary function [9].

The rarity of pituitary metastasis and the lack of specific clinical/radiological features hinder their differentiation from other sellar lesions, particularly in the absence of a history of cancer.

Our clinical case focused on the diagnosis of a pituitary metastasis in a patient without history of previous or active malignancy. We can speculate that in cases of aggressive pituitary lesions with a sudden onset, pituitary metastasis should be considered in the differential diagnosis.

Pituitary MRIs usually reveal a nonhomogenous invasive sellar mass, which sometimes appears as a dumbbell-shaped tumor, for the indentation of the diaphragm sellae [5]. The bone destruction may help distinguish pituitary metastases from a pituitary adenoma. The loss of the posterior lobe bright spot can also be observed [5]. Some studies have shown that magnetic resonance spectroscopy may be useful to distinguish pituitary metastases from pituitary adenomas, in cases of lesion smaller than 20 millimeters, according to neuroimaging data on cell proliferation and bleeding [7].

Functional imaging with [18F]-fluoro-d-glucose (18F-FDG) seem not able to certainly differentiate pituitary metastasis from pituitary adenomas. A positive 18F-FDG uptake has been reported in over 60% of macroadenomas. Interestingly, 2-^18^F-fluoroethyl-l-tyrosine was recognized useful to identify a pituitary metastasis [10].

There is lack of consensus on the treatment of patients with pituitary metastases. Chemotherapy may represent the first line treatment in several cases. Targeted therapy and chemotherapy may be adopted according to the type of primary tumor and metastatic spread. Surgery is usually reserved for patients with neurological symptoms. The surgical resection of pituitary metastasis relieves neurological symptom but is not associated with increased survival [1]. A total resection through a transsphenoidal approach is technically challenging due to invasion of densely packed adjacent structures, such as the cavernous sinus, and due to high vascularization of the tumor mass [1].

Data of efficacy of radiotherapy are superimposable to those reached from surgery, with a better tolerance [1]. Radiotherapy may be preferred to surgery for palliative purposes, particularly in patients with multiple brain metastases.

In conclusion, our clinical case underlines the importance of considering pituitary metastasis in the diagnostic workflow of pituitary lesions. Pituitary metastasis can be suspected if a sellar mass occurs in a patient with oncological history and in patients with aggressive pituitary lesions, even without oncological history as in our case. Our clinical experience suggests that rare and aggressive pituitary mass, such as metastasis, should be managed in pituitary tumor centers of excellence, to facilitate a prompt diagnosis and safe management according to a multidisciplinary approach that involves oncologists, radiation oncologists, neurosurgeons, neuroendocrinologists, neuroradiologists, and neuropathologists.

## Learning Points

- Clinicians should consider the possibility of pituitary metastasis in patients with symptoms and signs that may suggest the presence of a pituitary mass, particularly in a patient with cancer history.
- Since pituitary metastasis can be the first manifestation of cancer, the diagnosis requires a multidisciplinary team.
- Hypopituitarism, particularly central hypoadrenalism, may occur in patients with pituitary metastasis and requires immediate diagnosis and treatment, in order to hinder adrenal crisis that can be a life-threatening emergency.
- Prognosis is usually poor and it is difficult to estimate, as it varies significantly according to tumor staging, primary histology, and grade.
- Recognition and management of pituitary metastasis remains complex and requires a multidisciplinary team of endocrinologists, oncologists, neuropathologists, neurosurgeons, and neuroradiologists.

## Data Availability

Original data generated and analyzed during this study are included in this published article.

## Abbreviations

ACTH: adrenocorticotropic hormone
CK: cytokeratin
CT: computed tomography
FSH: follicle-stimulating hormone
LH: luteinizing hormone
TSH: thyrotropin (thyroid-stimulating hormone)
